# Performance of a Global Functional Assay Based on Interferon-γ Release to Predict Infectious Complications and Cancer After Kidney Transplantation

**DOI:** 10.3389/ti.2024.13551

**Published:** 2024-10-30

**Authors:** Mario Fernández-Ruiz, Tamara Ruiz-Merlo, Isabel Rodríguez-Goncer, José María Caso, Francisco López-Medrano, Patricia Parra, Rafael San Juan, Natalia Polanco, Esther González, Amado Andrés, José María Aguado, Natalia Redondo

**Affiliations:** ^1^ Unit of Infectious Diseases, Hospital Universitario “12 de Octubre”, Instituto de Investigación Sanitaria Hospital “12 de Octubre” (imas12), Madrid, Spain; ^2^ Centro de Investigación Biomédica en Red de Enfermedades Infecciosas (CIBERINFEC), Instituto de Salud Carlos III, Madrid, Spain; ^3^ Department of Medicine, School of Medicine, Universidad Complutense, Madrid, Spain; ^4^ Department of Nephrology, Hospital Universitario “12 de Octubre”, Instituto de Investigación Sanitaria Hospital “12 de Octubre” (imas12), Madrid, Spain

**Keywords:** kidney transplantation, infection, immune monitoring, functional, prediction

## Abstract

The QuantiFERON-Monitor assay (QTF-Monitor) is intended to assess innate and adaptive immune responses by quantifying interferon (IFN)-γ release upon whole blood stimulation with a TLR7/8 agonist and an anti-CD3 antibody. We performed the QTF-Monitor in 126 kidney transplant recipients (KTRs) at different points during the first 6 post-transplant months. The primary outcome was overall infection, whereas secondary outcomes included bacterial infection, opportunistic infection and *de novo* cancer. The association between IFN-γ production and outcomes was analyzed as “low” immune responses (<15 IU/mL) and as a continuous variable to explore alternative thresholds. There were no significant differences in the occurrence of overall infection according to the QTF-Monitor at any monitoring point. Regarding secondary outcomes, KTRs with a low response at week 2 experienced a higher incidence of bacterial infection (50.8% versus 24.4%; *P*-value = 0.006). Low response at month 1 was also associated with opportunistic infection (31.6% versus 14.3%; *P*-value = 0.033). The discriminative capacity of IFN-γ levels was poor (areas under the ROC curve: 0.677 and 0.659, respectively). No differences were observed for the remaining points or post-transplant cancer. In conclusion, the QTF-Monitor may have a role to predict bacterial and opportunistic infection in KTRs when performed early after transplantation.

## Graphical Abstract

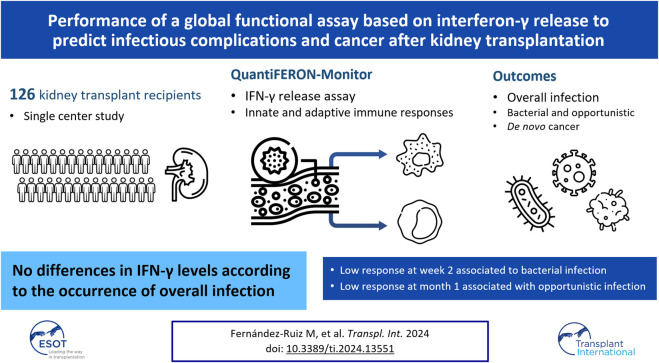

## Introduction

The excellent results achieved with current immunosuppressive regimens in terms of graft function and patient survival after solid organ transplantation (SOT) are threatened by the development of complications such as infections or cancer [1, 2]. Therefore, the discovery and validation of biomarkers capable of informing on the net state of immunosuppression constitutes a research priority [3]. Many of these assays are designed to quantify the adaptive response against specific pathogens, typically cytomegalovirus (CMV) [4]. In addition, some non-pathogen-specific parameters have been proven to predict the occurrence of post-transplant infection [5] or cancer [6]. None of these approaches, however, provides a comprehensive assessment of the functionality of the innate and adaptive components of the immune system.

The innate immunity is triggered through various families of pattern recognition receptors (PRRs) that detect distinct evolutionarily conserved structural motifs present in microorganisms. Toll-like receptors (TLRs) are central actors in the orchestration of the innate immunity and its interplay with the adaptive arm [7]. The activation of TLR signaling pathways leads to the transcriptional upregulation of genes involved in inflammatory responses, such as proinflammatory cytokines or type I interferons (IFNs) [8]. Research efforts have been focused on the effect exerted by immunosuppressive agents on T-cell and B-cell responses, given their role in allorecognition and graft rejection. In addition, a renewed interest has emerged on the contribution of innate responses to post-transplant events [9]. Multiple studies have shown that polymorphisms in genes encoding for PRRs modulate individual susceptibility to bacterial, viral and fungal pathogens [10–12]. It may be hypothesized that the relative contribution of innate immunity to the host defense becomes more evident upon abrogation of adaptive responses by long-term immunosuppression.

The QuantiFERON-Monitor (QTF-Monitor) is a commercial enzyme-linked immunosorbent assay (ELISA)-based IFN-γ release assay (IGRA) intended to quantify innate and adaptive immune responses following incubation of heparinized whole blood with an agonist of TLR7/8 (R848 or resiquimod) and an anti-CD3 monoclonal antibody [13]. Despite the advantages of this comprehensive approach, only a few studies have investigated the usefulness of QTF-Monitor to predict infectious complications after KT [14, 15], liver transplantation (LT) [16] or lung transplantation (LuT) [17]. In addition, no previous studies have evaluated the potential application of this assay to evaluate the risk of *de novo* malignancies after transplantation. The pathogenesis of this complication is multifactorial, with the participation of host (older age, sun exposure, pre-transplant history of cancer, smoking and alcohol consumption, latent infection by oncogenic viruses) and transplant-related factors (such as donor-transmitted cancer) [18]. Nevertheless, the deleterious effect of immunosuppressive therapy on cancer immune surveillance and the assumed concept that post-transplant cancer acts as a marker of over-immunosuppression provide the rationale to investigate whether an assay able to interrogate innate and adaptive responses may be also useful to predict the occurrence of malignancy.

With these research gaps in mind, we have assessed the functional immune status of a single-center cohort of KT recipients by means of the QTF-Monitor assay performed at multiple points throughout the first 6 months in order to characterize the dynamics of IFN-γ levels and their correlation with the development of infection and cancer.

## Patients and Methods

### Study Design and Setting

We included consecutive adult patients that underwent KT at our institution between February 2018 and July 2019. Patients experiencing primary graft non-function or early (first week) graft loss were excluded. All participants provided written informed consent at study entry, which was carried out in accordance with the ethical standards laid down in the Declarations of Helsinki and Istanbul. The study protocol was approved by the local Clinical Research Ethics Committee (reference 14/030).

All the participants were prospectively followed-up for at least 12 months, unless graft loss or death occurred earlier. Immunosuppression and prophylaxis regimens are described in Supplementary Methods. A number of pre-transplant, transplant-related and post-transplant variables were collected by means of a standardized case report form.

The QTF-Monitor assay was performed at week 2 (±4 days) and months 1, 3, and 4 (± 1 week) and 6 (±3 weeks). Peripheral blood lymphocyte subpopulations (CD3^+^, CD4^+^, and CD8^+^ T-cell counts) were assessed at months 1, 3, and 6 with an automated multicolor flow cytometry system (BD Multitest™ six-color TBNK reagent with acquisition on the BD FACSCanto II instrument using BD FACSCanto clinical software, all from BD Biosciences, San Jose, CA).

### Study Outcomes

The *primary study outcome* was the incidence of post-transplant infection during the follow-up according to the functional immune competence (low versus moderate or high responses) as assessed by the QTF-Monitor assay. As *secondary outcomes* we separately analyzed the incidence of bacterial and opportunistic infection, as well as post-transplant *de novo* malignancy. For those outcomes for which a significant association with the presence of a low response (as defined by the manufacturer) was observed, alternative cut-off values for IFN-γ levels were explored on the basis of the best combination of sensitivity and specificity, as detailed below. Finally, as an additional secondary outcome we investigated the clinical variables that were associated with a low immune response at the different times after transplantation.

### Procedure for the QTF-Monitor Assay

The QTF-Monitor assay (Qiagen GmbH, Hilden, Germany) was performed according to manufacturer’s recommendations. Whole blood samples were obtained by venipuncture in lithium heparin vacuum blood collection tubes, stored at room temperature and processed within less than 6 h. 1-mL aliquots were transferred to the QTF-Monitor blood collection tubes for stimulation and incubation. The QTF-Monitor lyophilized stimulants (LyoSpheres) containing the immune ligands anti-CD3 and R848 were equilibrated to room temperature, and one LyoSphere was transferred to the blood collection tube, which was gently shaken 5–10 times to ensure complete dissolution. The QTF-Monitor tubes were immediately placed into a 37°C incubator for 16–24 h. After incubation, plasma was harvested by centrifugation at 2,000 to 3,000 × g for 15 min, and stored at −80°C until analysis. The amount of IFN-γ produced was quantified in undiluted and diluted (1:10 and 1:100) plasma samples by means of the QTF-Monitor ELISA kit and given as international units (IU)/mL by means of the QTF-Monitor Analysis Software (all from Qiagen). The lyophilized IFN-γ standard was reconstituted with distilled water to prepare the standard curve. All these procedures were performed by a single technician that was blind to patient characteristics. Results were interpreted according to the cut-off values for IFN-γ proposed in the package insert: low (<15 IU/mL), moderate (15–1,000 IU/mL) and high (>1,000 IU/mL) immune responses.

### Study Definitions

The diagnosis of post-transplant infection was based on microbiological findings in association with a compatible clinical syndrome according to the definitions proposed by the Centers for Disease Control and Prevention’s National Healthcare Safety Network (CDC/NHSN) [19]. Febrile episodes with no microbiological documentation that resolved spontaneously without antimicrobial treatment were excluded, as were asymptomatic bacteriuria and lower urinary tract infection. The diagnosis of CMV disease (viral syndrome or end-organ disease) required the demonstration of CMV replication by real-time PCR in the presence of attributable symptoms [20]. Opportunistic infection was operationally defined according to previous studies [21, 22] and included tuberculosis, listeriosis, infections due to facultatively intracellular bacteria (e.g., *Rhodococcus),* herpes simplex virus and varicella-zoster virus (shingles), proven/presumptive BK polyomavirus-associated nephropathy (BKPyVAN) [23], proven/probable invasive fungal disease (IFD) [24], *Pneumocystis jirovecii* pneumonia, toxoplasmosis and visceral leishmaniasis. The diagnosis of *de novo* cancer required histological confirmation and the absence of a pre-transplant history of such malignancy (i.e., type and site). Additional definitions are provided in Supplementary Methods.

### Statistical Analysis

Quantitative data were expressed with the mean ± standard deviation (SD) or the median with interquartile range (IQR). Categorical variables were compared with the χ^2^ test. Student’s t-test or *U* Mann-Whitney test were applied for continuous variables. Repeated QTF-Monitor results within the same patient were compared with the Wilcoxon test, whereas paired proportions were compared with the McNemar test. Correlations were assessed using either Pearson’s r or Spearman’s rho. The association between the QTF-Monitor assay at each point and subsequent outcomes was explored by stratifying IFN-γ levels as per the interpretative cut-off values offered in the assay package insert (low versus moderate-high responses). Alternative cut-off values were subsequently evaluated for those primary or secondary outcomes depicting significant associations in the previous approach by means of the Youden’s J statistic, which combines sensitivity and specificity into a single measure (J = sensitivity + specificity − 1). The discriminative capacity of IFN-γ levels analyzed as a continuous variable was explored with the area under the receiving operating characteristic (auROC) curve. We estimated the diagnostic accuracy (sensitivity, specificity, positive [PPV] and negative predictive values [NPV] with the corresponding 95% confidence intervals [CIs]). Time-to-event curves were plotted by the Kaplan-Meier method and inter-group differences were compared with the log-rank test. IFN-γ levels were log_10_-transformed for statistical analyses. Statistical analysis was performed with SPSS version 29.0.1.0 (IBM Corp., Armonk, NY).

## Results

### Clinical Characteristics

The study cohort comprised 126 KT recipients (Table 1). The QTF-Monitor assay was performed at 439 different instances, with a median of 4 (IQR: 3–4) measurements per patient. In detail, the assay was available for 112 patients at week 2 (91.1% of those that survived with a functioning graft at that point), 108 patients at month 1 (87.8%), 67 patients at month 3 (54.9%), 52 patients at month 4 (42.9%), and 100 patients at month 6 (82.6%).

**TABLE 1:** Demographics and clinical characteristics of the study cohort (n = 126).

Variable	
Age of recipient, years [mean ± SD]	54.9 ± 15.5
Male gender of recipient [n (%)]	83 (65.9)
Prior or current smoking history [n (%)]	48 (38.1)
BMI at transplantation, Kg/m^2^ [mean ± SD]^a^	25.4 ± 4.3
Pre-transplant chronic comorbidities [n (%)]	
Hypertension	100 (79.4)
Diabetes mellitus	38 (30.2)
Other chronic heart disease	17 (13.5)
Coronary heart disease	13 (10.3)
Chronic pulmonary disease	11 (8.7)
Cerebrovascular disease	7 (5.6)
Peripheral arterial disease	4 (3.2)
Previous kidney transplantation [n (%)]	27 (21.4)
Underlying end-stage renal disease [n (%)]	
Glomerulonephritis	29 (23.0)
Diabetic nephropathy	32 (25.4)
Polycystic kidney disease	11 (8.7)
Nephroangiosclerosis	9 (7.1)
Chronic interstitial nephropathy	8 (6.3)
Loss of renal mass and hyperfiltration injury	6 (4.8)
Reflux nephropathy	5 (4.0)
Lupus nephropathy	4 (3.2)
Congenital nephropathy	5 (4.0)
Unknown	10 (7.9)
Other	7 (5.6)
CMV serostatus [n (%)]	
D+/R+	74 (58.7)
D−/R+	26 (20.6)
D+/R−	24 (19.0)
D unknown/R+	1 (0.8)
D-/R-	1 (0.8)
Positive EBV serostatus (anti-EBNA IgG) [n (%)]^b^	115 (91.3)
Positive HCV serostatus [n (%)]	7 (5.6)
Positive HBsAg status [n (%)]	4 (3.2)
Positive HIV serostatus [n (%)]	3 (2.4)
Pre-transplant renal replacement therapy [n (%)]	110 (87.3)
Hemodialysis	85/110 (67.5)
Continuous ambulatory peritoneal dialysis	25/110 (19.8)
Time on dialysis, months [median (IQR)]	23.1 (12.9–46.8)
Type of transplantation [n (%)]	
Single kidney	118 (93.7)
Double kidney	2 (1.6)
Simultaneous pancreas-kidney	6 (4.8)
Age of donor, years [mean ± SD]	53.4 ± 17.0
Male gender of donor [n (%)]	66 (52.4)
Type of donor [n (%)]	
DBD donor	78 (61.9)
Uncontrolled DCD donor (Maastricht categories 1–2)	11 (8.7)
Controlled DCD donor (Maastricht categories 3–4)	12 (9.5)
Living donor	25 (19.8)
Cold ischemia time, hours [mean ± SD]	
Number of HLA mismatches [median (IQR)]	4 (3–5)
Induction therapy [n (%)]	
Antithymocyte globulin	59 (46.8)
Basiliximab	57 (45.2)
None	10 (7.9)
Primary immunosuppression regimen [n (%)]	
Prednisone, tacrolimus and MMF/MPS	111 (88.1)
Prednisone, tacrolimus and everolimus	10 (7.9)
Prednisone, tacrolimus and azathioprine	5 (4.0)
CMV prevention strategy [n (%)]	
Antiviral prophylaxis with VGCV	75 (59.5)
Duration of prophylaxis, days [median (IQR)]	111 (91–183)
Preemptive therapy	51 (40.5)
Follow-up, days [median (IQR)]	532 (480–727)
Post-transplant complications at 1 year [n (%)]	
Delayed graft function	45 (35.7)
Number of dialysis sessions [median (IQR)]	2 (1–4)
Development of *de novo* DSA	8 (6.3)
Surgical reintervention within the first month	18 (14.3)
Renal artery stenosis	14 (11.1)
New-onset diabetes	11 (8.7)
Atherothrombotic event	2 (1.6)
Biopsy-proven acute graft rejection	12 (9.5)
Time from transplantation, days [median (IQR)]	86 (14.8–154.5)
T-cell-mediated rejection	6 (4.8)
Borderline T-cell-mediated rejection	5 (4.0)
Antibody-mediated rejection	1 (0.8)

BMI, body mass index; CMV, cytomegalovirus; D, donor; DBD, donation after brain death; DCD, donation after circulatory death; DSA, donor-specific antibody; EBV, Epstein-Barr virus; EBNA, EBV nuclear antigen; HLA, human leukocyte antigen; HBsAg, hepatitis B virus surface antigen; HCV, hepatitis C virus; HIV, human immunodeficiency virus; IQR, interquartile range; MMF/MPS, mycophenolate mofetil/enteric-coated mycophenolate sodium; R, recipient; SD, standard deviation; VGCV, valganciclovir.

^a^ Data on BMI not available for 25 patients.

^b^ Data on EBV serostatus not available for 4 patients.

### Post-Transplant Kinetics and Clinical Determinants of IFN-γ Production

**FIGURE 1:**
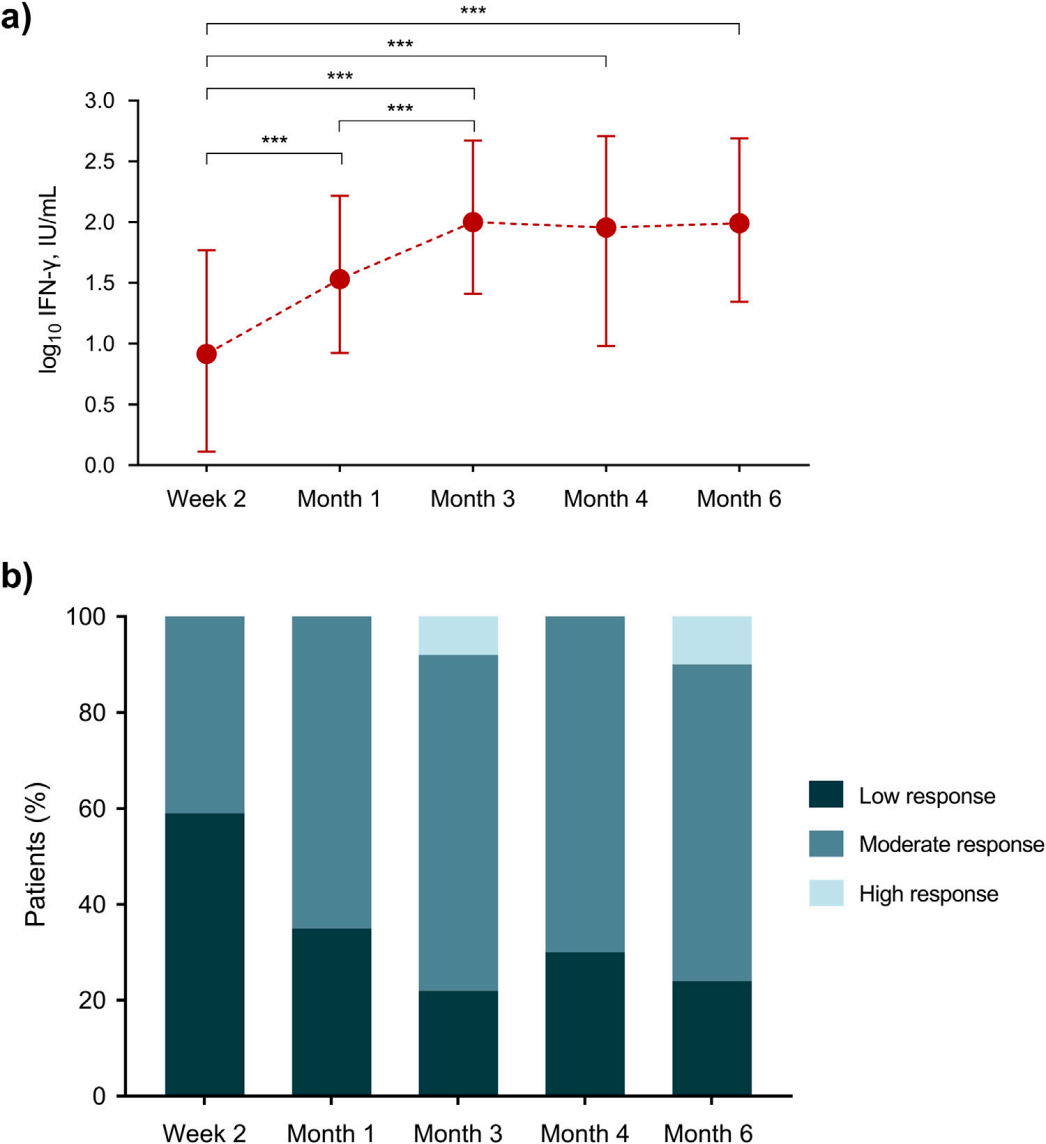
**(A)** Kinetics of IFN-γ levels measured by the QTF-Monitor assay; points and bars show the median and interquartile range, respectively. **(B)** Proportion of patients with different immune responses according to the cut-off values for IFN-γ production proposed by the manufacturer [low (<15 IU/mL), moderate (15–1,000 IU/mL) and high (>1,000 IU/mL)]. *** *P*-value for repeated measures <0.0001. IFN-γ: interferon-γ; IU: international unit.

Overall, median IFN-γ levels showed a significant increase from week 2 [0.9 (IQR: 0.1–1.8) log_10_ IU/mL] to month 1 [1.5 (IQR: 0.9–2.2) log_10_ IU/mL; *P*-value < 0.0001] and month 3 [1.9 (IQR: 1.4–2.7) log_10_ IU/mL; *P*-value < 0.0001], to reach a plateau beyond that point. In accordance, the proportion of patients with a low immune response (<15 IU/mL) decreased from week 2 [58.9% (66/112)] to month 6 [24.0% (24/100); *P*-value < 0.0001] (Figure 1).

We explored the clinical variables predictive of a low immune response. Pre-transplant dialysis [93.9% (62/66) versus 78.3% (36/46); *P*-value = 0.014], induction therapy with antithymocyte globulin (ATG) [59.1% (39/66) versus 28.3% (13/46); *P*-value = 0.001] and delayed graft function [45.5% (30/66) versus 21.7% (10/46); *P*-value = 0.016] were more common in KT recipients exhibiting a low response at week 2 (Supplementary Table S1). The associations with pre-transplant dialysis and ATG induction were also observed for the results of the assay at month 1. Living donation was less likely in recipients with low responses at that point [5.3% (2/38) versus 27.1% (19/70); *P*-value = 0.006]. In addition, absolute lymphocyte and CD3^+^ and CD4^+^ T-cell counts were lower in this group (Supplementary Table S2). No significant associations were found between clinical features or laboratory values and the assay results at month 6 (Supplementary Table S3).

**FIGURE 2:**
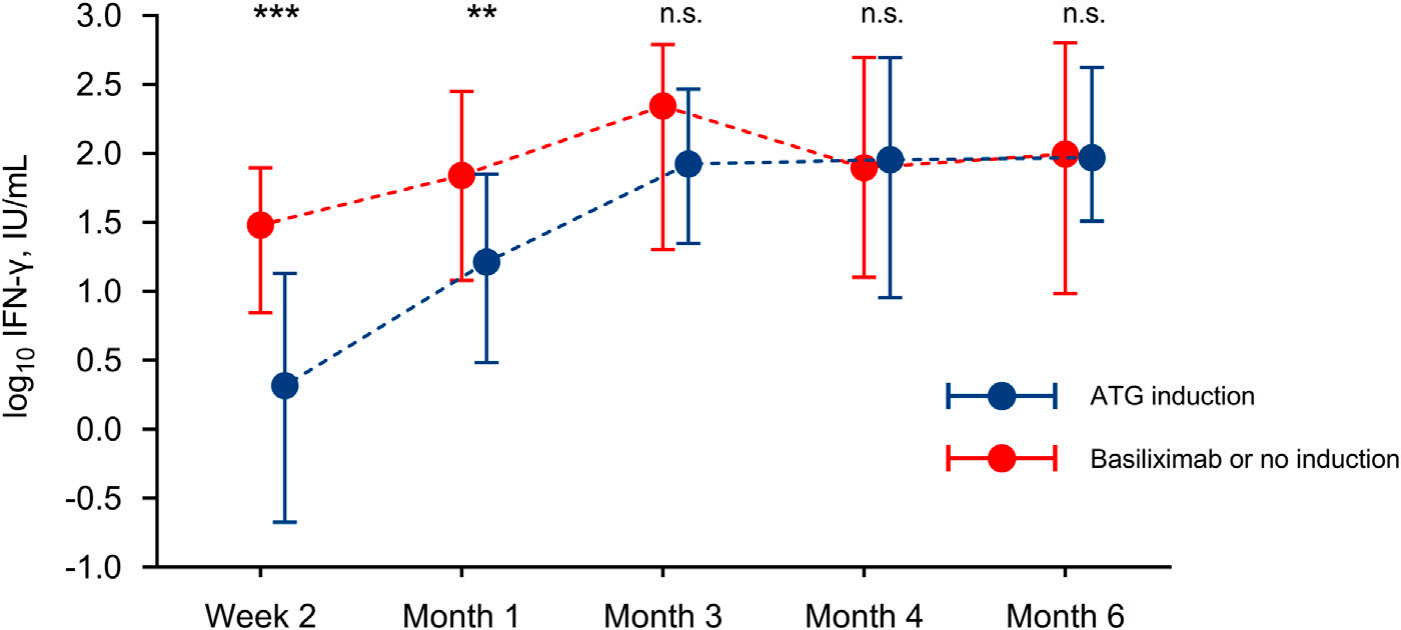
Kinetics of IFN-γ levels according to the administration of induction therapy with ATG; points and bars show the median and interquartile range, respectively. ** *P*-value < 0.001, *** *P*-value < 0.0001. ATG, antithymocyte globulin; IFN-γ, interferon-γ; IU, international unit; ns, not significant.

To further investigate the effect of induction therapy, we analyzed IFN-γ levels as a continuous variable. Patients treated with ATG showed a significantly lower production of IFN-γ at week 2 and month 1 as compared to those that received basiliximab or no induction (Figure 2). In accordance with the lymphocyte-depleting effect of ATG, a significant correlation was observed between IFN-γ levels and CD3^+^, CD4^+^ and CD8^+^ T-cell counts at month 1 (but not at months 3 or 6), with Spearman’s Rho coefficients ranging from 0.346 to 0.378 (Supplementary Figure S1).

We also investigated whether IFN-γ production was correlated with concurrent measurements of tacrolimus trough levels. We only found a weak inverse correlation at month 6 after transplantation (Pearson’s r: −0.338; *P*-value = 0.010), whereas no correlations were observed for week 2 (r: −0.181; *P*-value = 0.152), month 1 (r: −0.001; *P*-value = 0.993), month 3 (r: −0.049; *P*-value = 0.771) or month 4 (r: 0.033; *P*-value = 0.876).

### Post-Transplant Infection and Cancer

Overall, 72 patients (57.1%) experienced 145 episodes of post-transplant infection (primary outcome). The median interval to the first episode was 83.5 days (IQR: 26.5–227.8). Acute graft pyelonephritis [51 episodes (35.2%)] and pneumonia [17 (11.7%)] were the most common types. Enterobacterales accounted for most of the microbiologically documented cases, with predominance of *Escherichia coli* [33 episodes (22.7%)] and *Klebsiella pneumoniae* [24 (16.5%)] (Supplementary Table S4).

Regarding secondary outcomes, 50 patients (39.7%) were diagnosed with 105 episodes of bacterial infection [median interval to the first episode of 64.5 days (IQR: 17.8–196)]. On the other hand, 28 episodes in 26 patients (20.6%) met the definition of opportunistic infection [median interval of 167.5 days (IQR: 82.8–295.8)], with CMV disease [12 episodes (42.9%)] and herpes zoster [6 (21.4%)] as the most common forms (Supplementary Table S5). Eleven patients (8.7%) developed *de novo* cancer at a median of 364 days (IQR: 169.5–594). In detail, there were six cases of non-melanoma skin cancer and six cases of solid cancer (one patient had both) (Supplementary Table S6).

### Association Between the Functionality of Immune Response and Overall Post-transplant Infection

**FIGURE 3:**
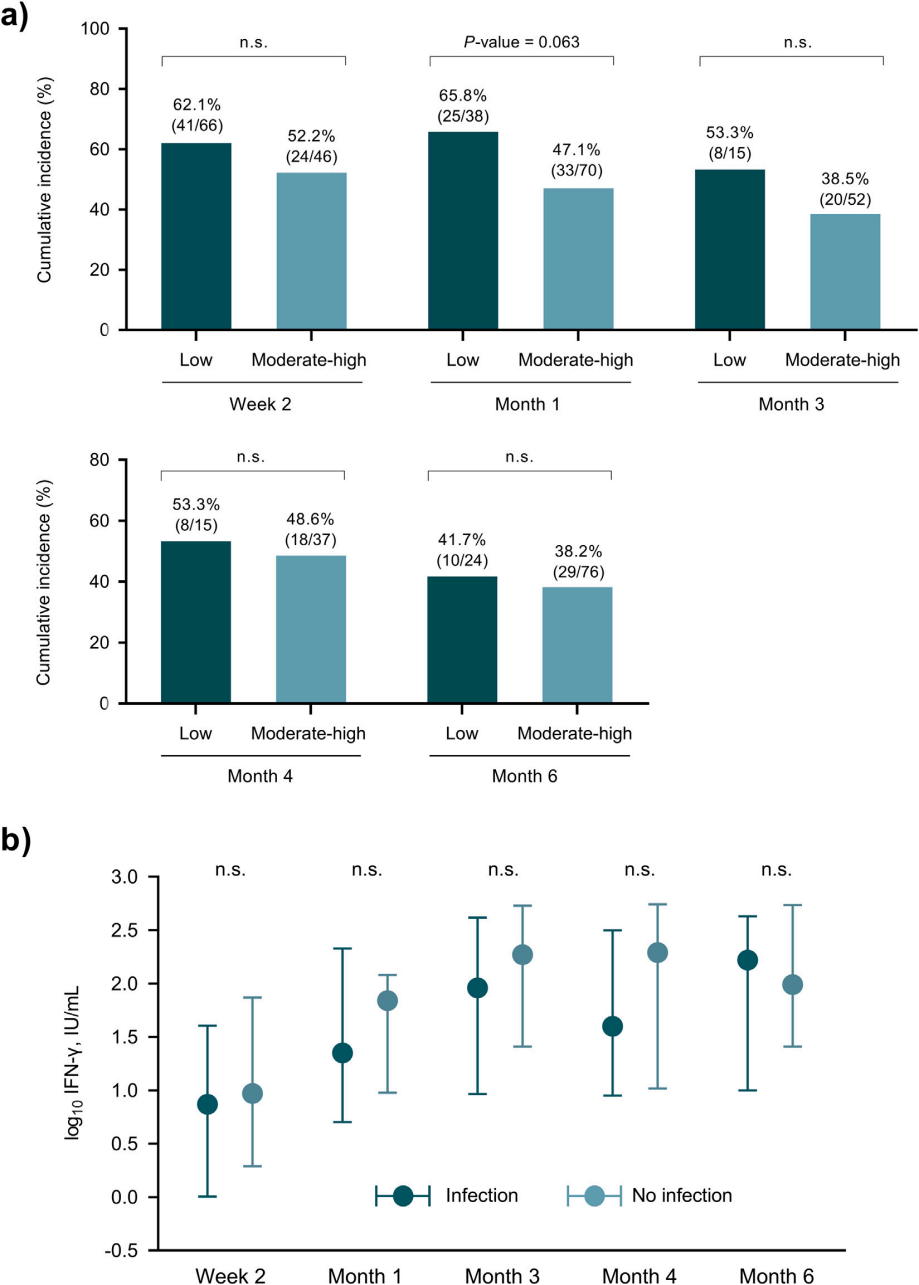
**(A)** Cumulative incidence of post-transplant infection (primary outcome) according to the immune response in the QTF-Monitor assay at different time points after transplantation. **(B)** IFN-γ levels according to the subsequent occurrence of infection; points and bars show the median and interquartile range, respectively. ns, not significant; IU, international unit.

There were no significant differences in the cumulative incidence of overall infection between KT recipients exhibiting a low immune response (IFN-γ <15 IU/mL) and those with a moderate or high response at each monitoring point. We only found a non-significant trend towards a higher risk among patients with low responses at month 1 [65.8% (25/38) versus 47.1% (33/70); *P*-value = 0.063] (Figure 3A). There were no significant differences in IFN-γ levels (taken as a continuous variable) between patients with or without infection (Figure 3B).

As a measure of sustained over-immunosuppression, we compared the incidence of infection between KT recipients with responses categorized as low in all the assays performed throughout the first post-transplant months and the rest of the cohort. There were no significant differences for persistent low responses either during the first 3 [45.5% (10/22) versus 43.0% (43/100); *P*-value = 1.000] or 6 months [33.3% (6/18) versus 35.9% (37/103), respectively; *P*-value = 1.000].

### Association Between the Functionality of Immune Response and Secondary Outcomes

**FIGURE 4:**
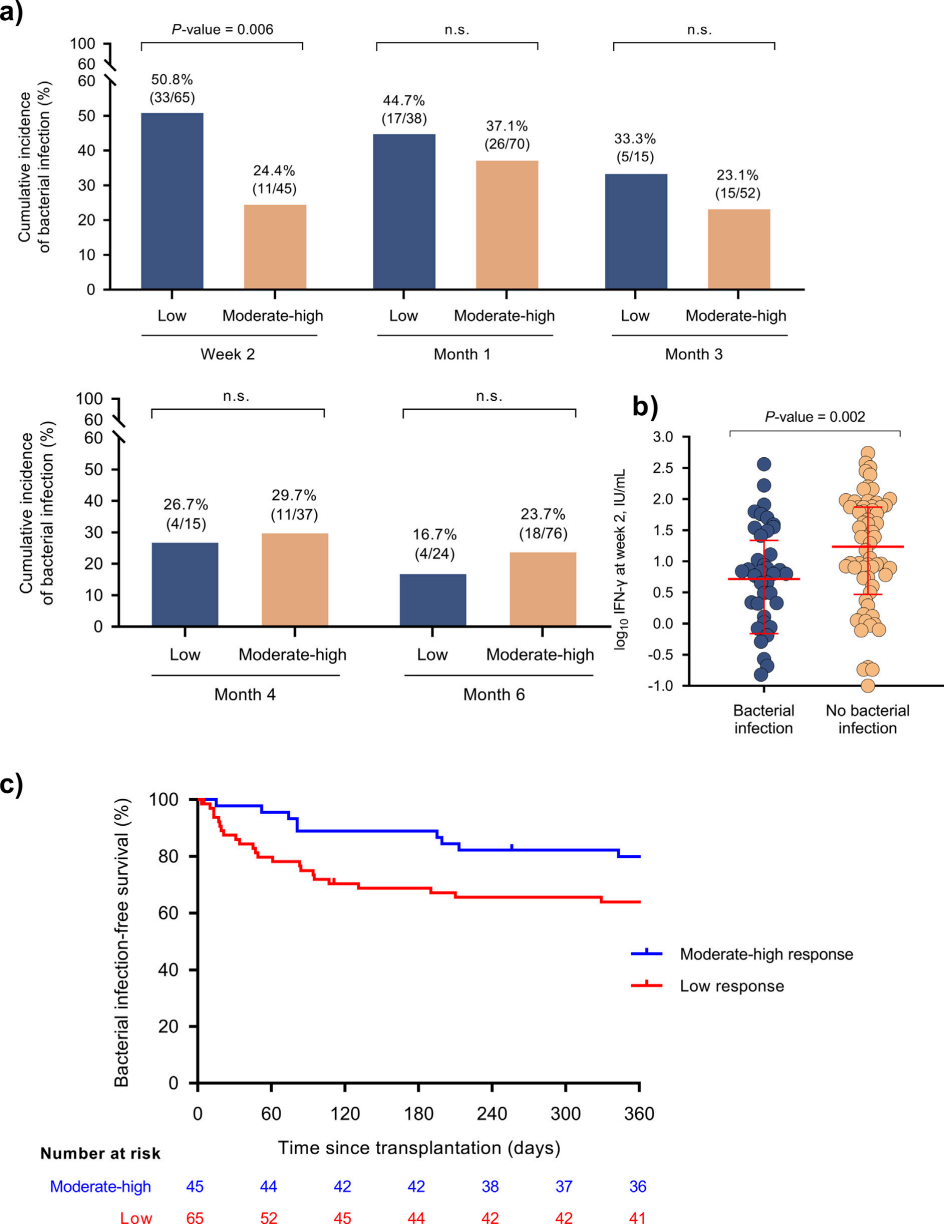
**(A)** Cumulative incidence of post-transplant bacterial infection (secondary outcome) according to the immune response in the QTF-Monitor assay at different time points after transplantation. **(B)** IFN-γ levels at week 2 according to the subsequent occurrence of bacterial infection; points and bars show the median and interquartile range, respectively. **(C)** Bacterial infection-free survival according to the immune response at week 2 (log-rank test *P*-value = 0.009). IFN-γ, interferon-γ; IU, international unit.

Patients with a low response at the early (2-week) assessment had a higher cumulative incidence of bacterial infection than those with an intermediate response [50.8% (33/65) versus 24.4% (11/45), respectively; *P*-value = 0.006] (Figure 4A). IFN-γ production at week 2 was accordingly lower among patients developing bacterial infection (Figure 4B). One-year bacterial infection-free survival was significantly lower in the presence of a low response (Figure 4C). On the contrary, there were no differences for the remaining points in terms of the magnitude of response (low versus intermediate-high) (Figure 4A; Supplementary Table S7) or the absolute IFN-γ level (Supplementary Table S8).

**FIGURE 5:**
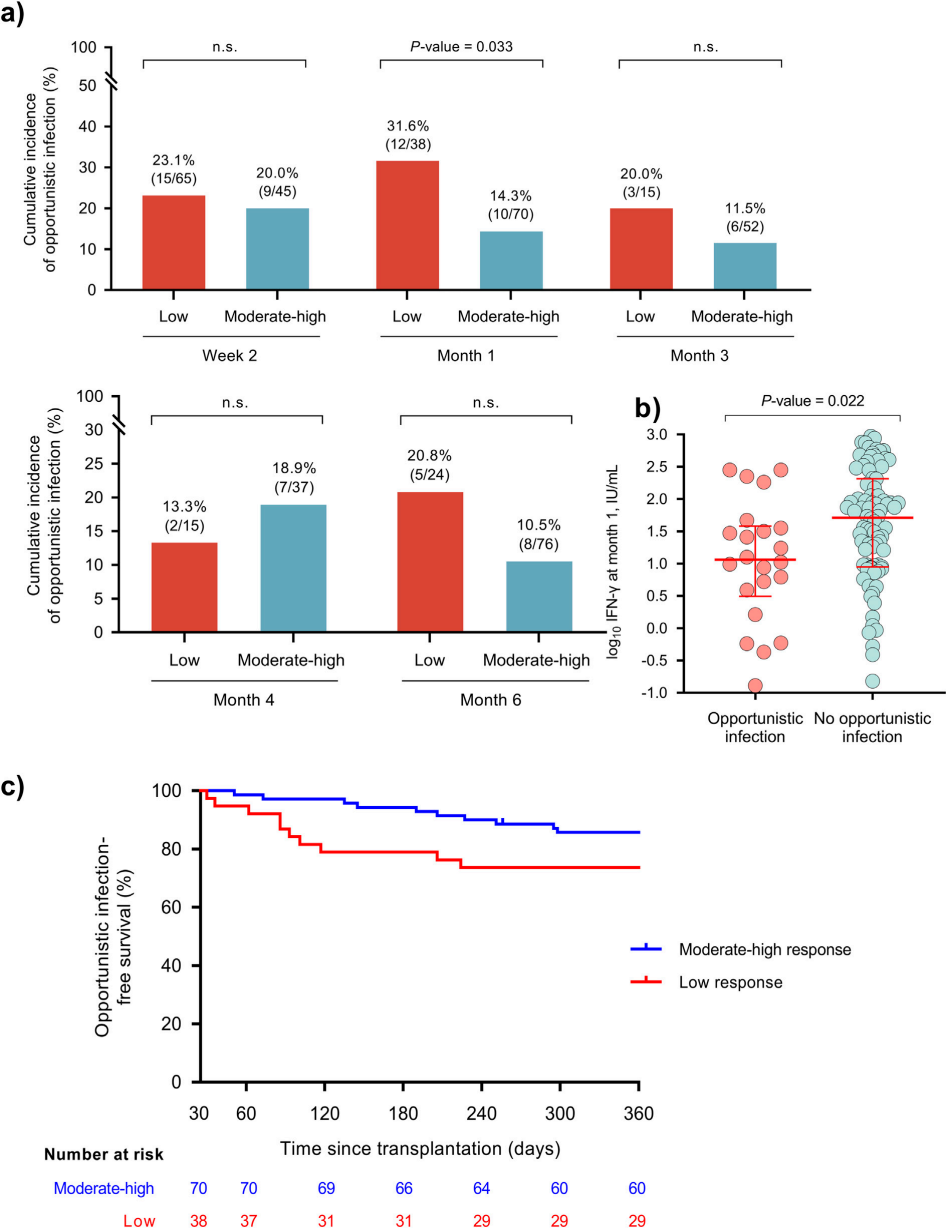
**(A)** Cumulative incidence of post-transplant opportunistic infection (secondary outcome) according to the immune response in the QTF-Monitor assay at month 1. **(B)** IFN-γ levels at month 1 according to the subsequent occurrence of bacterial infection; points and bars show the median and interquartile range, respectively. **(C)** Opportunistic infection-free survival according to the immune response at week 2 (log-rank test *P*-value = 0.026). IFN-γ, interferon-γ; IU, international unit.

Regarding opportunistic infection, the presence of a low response at month 1 was associated with the subsequent development of this secondary outcome [31.6% (12/38) versus 14.3% (10/70); *P*-value = 0.033] (Figure 5A). The IFN-γ level at this point was also lower in patients developing opportunistic infection (Figure 5B), as was the 1-year event-free survival in patients with a low response (Figure 5C). No differences were observed for the remaining time points (Supplementary Tables S7 and S8).

Finally, there were no differences in the incidence of *de novo* malignancy according to the functionality of immune responses (Figure 5A; Supplementary Table S7) or IFN-γ levels (Supplementary Table S8).

### Diagnostic Accuracy of the QTF-Monitor Assay to Predict Bacterial and Opportunistic Infection

In view of the associations found at the early assessment, we further explored the diagnostic accuracy for the secondary outcomes of bacterial and opportunistic infection. By applying the cut-off value proposed by the manufacturer (IFN-γ <15 IU/mL), we obtained a sensitivity of 75.0% (95% CI: 59.7–86.8) and specificity of 51.5% (95% CI: 38.9–64.0) to predict bacterial infection beyond week 2. The corresponding values for the development of opportunistic infection beyond month 1 were 54.6% (95% CI: 32.2–75.6) and 69.8% (95% CI: 58.9–79.2), respectively (Supplementary Table S9). The discriminative capacity of IFN-γ levels was overall low, with auROCs for predicting bacterial and opportunistic infection of 0.677 (95% CI: 0.576–0.778) and 0.659 (95% CI: 0.539–0.779), respectively.

We also explored alternative cut-off values according to the optimal balance between sensitivity and specificity. By using a threshold at week 2 of 7.9 IFN-γ IU/mL, the 1-year bacterial infection-free survival curve of patients with low response was more clearly separated from those above the cut-off (Supplementary Figure S2), yielding improved specificity [66.7% (95% CI: 53.9–77.8)] and PPV [57.7% (95% CI: 47.8–66.9)] at the expense of a loss of sensitivity [68.2% (95% CI: 52.4–81.4)]. On the other hand, the optimal cut-off value to predict opportunistic infection beyond month 1 was set at 47.3 IU/mL, which also resulted in more clearly separated even-free survival curves (Supplementary Figure S3). As compared to the manufacturer’s criterion, this alternative cut-off resulted in improved sensitivity [81.8% (95% CI: 59.7–94.8)] and NPV [91.7% (95% CI: 81.6–96.5)], but poorer specificity (51.2% 95% CI: 40.1–62.1) (Supplementary Table S9).

## Discussion

Most of the biomarkers proposed to determine the net state of immunosuppression after SOT share two limitations: the lack of functional measurements ―as is the case with immunoglobulin levels or lymphocyte counts [3]― and the sole interrogation of virus-specific adaptive responses [4]. The QTF-Monitor assay offers the possibility of a broader functional assessment by measuring IFN-γ release upon *in vitro* stimulation of the innate and adaptive arms [13]. In the present experience the assay’s performance was moderate at best, since no association could be demonstrated between IFN-γ production (either categorized as “low” immune responses or as a continuous variable) at different points during the first 6 months and the primary outcome of overall infection. Nevertheless, we found that the QTF-Monitor results obtained during the first weeks may still be valuable to specifically predict the occurrence of bacterial or opportunistic infection, although this finding should be taken with caution due to the non-negligible false positive risk in the assessment of secondary outcomes. On the other hand, no apparent associations were found for *de novo* cancer.

The performance of the QTF-Monitor assay to predict post-transplant infection has been investigated by a few groups, with variable reported accuracy [14–17]. In a mixed cohort of 137 SOT recipients, Mian et al. observed that IFN-γ levels measured between months 1 and 6 were significantly lower in patients that developed subsequent infection and proposed an optimal threshold of ≤10 IU/mL. Urinary tract infection and pneumonia were the most common syndromes during the early post-transplant period, with a shift to predominance of viral pathogens beyond month 3. No multivariate analysis was performed to confirm the predictive value of IFN-γ production [14]. In contrast, a cross-sectional study at a mean of 2.6 post-transplant years failed to show differences in IFN-γ levels between stable KT recipients and those with infection. A subgroup analysis revealed that patients with bacterial infection had a significantly decreased IFN-γ release. Such an association, however, was not confirmed after adjustment for steroid dose and tacrolimus levels [15]. In a single-center cohort of LT recipients, IFN-γ levels at week 1 exhibited a fairly good capacity to predict infection through the first month, with the majority of the events being classified as opportunistic [16]. Finally, a recent study recruited 80 LuT recipients in which the QTF-Monitor was performed at 2, 6, 12, 24, and 52 weeks. The presence of IFN-γ levels <10 and <60 IU/mL at weeks 12 and 24, respectively, was associated with the diagnosis of opportunistic infection (mainly CMV viremia and IFD). Similar results were not observed for earlier monitoring points [17].

The discordant results from the existing literature, including those reported herein, may be partially attributable to differences across studies in outcomes and definitions, as well as in the timing and frequency of monitoring. Taken together, they would suggest that the QTF-Monitor assay may perform better for predicting some specific types of infection ―particularly of bacterial origin [15]― and when performed early after transplantation. Indeed, we have only identified differences in the assay results obtained at week 2 and month 1 according to the subsequent diagnosis of bacterial and opportunistic infection, respectively (with the latter mostly represented by CMV disease and herpes zoster). These results are in line with those previously observed among LT recipients [16]. Of note, the discriminative capacity for both outcomes was low, as indicated by auROC values below 0.700. Sood et al. reported a slightly better accuracy for the results obtained at week 1 after LT (auROC of 0.740) [16]. To put these findings into context, our group has reported higher discriminative capacities for other non-pathogen-specific biomarkers, such as the CD8^+^ T-cell count at month 1 (auROC of 0.739) or the total lymphocyte count at month 6 (auROC of 0.820) to predict opportunistic infection [25], Torque Teno virus (TTV DNAemia) at month 1 for predicting opportunistic infection and/or cancer (auROC of 0.704) [22], or serum sCD30 at month 1 for predicting bacterial infection (auROC of 0.846) [26]. Therefore, the potential contribution of the assay to the existing prediction models for post-transplant infection ―such as the externally validated SIMPLICITY Score [5]― should be explored in future studies.

Our results align with the cross-sectional study by Margeta et al [15] in that the performance of QTF-Monitor assay decreases at late periods after transplantation, once the amount of immunosuppression has been stabilized in most recipients. No differences in IFN-γ levels beyond month 1 were observed for any of the outcomes analyzed. Interestingly, we found no association between the QTF-Monitor results and the development of post-transplant cancer, a complication that usually results from the long-term effect of sustained over-immunosuppression [27]. No previous studies have investigated the role of QTF-Monitor assay to predict *de novo* malignancy. Although the number of events was low (n = 14), this negative finding would point to a lower relative contribution to the assay results of T-cell responsiveness (as compared to TLR-mediated innate responses), taken into account the pivotal role of cellular immunity in cancer immune surveillance. In contrast, we and others have shown that certain immune biomarkers assessed within the first months are useful to identify SOT recipients at increased risk of developing cancer in the mid- and long-term follow-up, such as CD4^+^ and CD8^+^ T-cell counts [6, 28], monocytic myeloid-derived suppressor cells [29] or TTV DNAemia [22].

The kinetics of IFN-γ levels measured by the QTF-Monitor assay was comparable to previous studies, which typically describe a sharp decline from the pre-transplant assessment followed by a progressive recovery through months 3–6 and a plateau thereafter [13, 14, 16, 17]. This pattern is in line with the accepted timing for immune reconstitution after SOT, as validated with other biomarkers such as TTV DNA load [30, 31]. The clinical factors influencing assay results have been only partially investigated. The association between the use of ATG as induction therapy and a lower IFN-γ production has been reported by other authors [14]. In our experience this effect persisted until month 1 and was supported by the inverse correlation observed between IFN-γ levels and T-cell counts. The impact of tacrolimus levels is less consistent, with studies reporting either strong [17] or borderline correlations [15], or even no apparent association [14]. We only found a weak inverse correlation with tacrolimus levels at month 6. Mian et al. also reported an association with daily doses of prednisone and mycophenolate [14], which were not recorded in our database. Although beyond the scope of our research, we found no significant association between the immune status measured by the QTF-Monitor assay at the different monitoring points and the subsequent occurrence of biopsy-proven acute rejection (data not shown). Patients with a low response at week 2 were more likely to have received pre-transplant dialysis and to have experienced delayed graft function (defined by the early requirement of renal replacement therapy). The deleterious effect of dialysis on the T-cell ability to produce IFN-γ after specific stimulation is well established for IGRAs used to detect *Mycobacterium tuberculosis* infection due to insufficient mitogen response and premature immune aging [32, 33]. Inversely, living donation was associated with a more robust immune response, which may be explained by the lower recipient age and the immediate graft function in this subgroup.

What may be the position of the QTF-Monitor assay for immune monitoring in the clinical arena? With the limitations inherent to multiple secondary outcome analyses and the lack of consistent associations at later points, our results would point out to the potential usefulness of the early assessment within the first weeks with the specific aim of predicting bacterial infection. By decreasing the IFN-γ threshold to <7.9 IU/mL we obtained a sensible improvement in specificity without a major impact in sensitivity, although the resulting estimates (66.7% and 68.2%, respectively) were far from excellent. Sood et al. proposed a clinical threshold of <1.30 IU/mL as the most discriminative to predict infection beyond the first week after LT, with a diagnostic accuracy in the line of our results (sensitivity of 71.4% and specificity of 63.0%) [16]. On the other hand, an alternative threshold (<47.3 IU/mL) at month 1 yielded a reasonable sensitivity (81.8%) to predict opportunistic infection, at the expense of a poor specificity (51.2%). Gardiner et al. also found a relatively low discriminative ability for different outcomes (overall infection, severe infection or opportunistic infection) and monitoring points after LuT [17]. In our opinion, any decision regarding the implementation of the QTF-Monitor assay in daily practice must balance diagnostic accuracy (which was found to be suboptimal in our experience), requirement of laboratory resources and economic costs with those of alternative biomarkers [3]. For instance, the observed impact on IFN-γ production of ATG induction and CD4^+^ and CD8^+^ T-cell counts would suggest that low responses may ultimately act as a surrogate for the presence of lymphocytopenia, which constitutes a well-established biomarker for opportunistic infection [25, 34–38].

Our study is based on a large cohort of KT recipients with regular monitoring, and it is strengthened by the assessment of immunosuppression-related complications which comprised infections and malignancies. We also provided an insight into the clinical determinants of the IFN-γ kinetics, including peripheral blood lymphocyte subpopulations. Nevertheless, a number of limitations must be noted, such as the relatively low number of some events, which may have limited statistical power. Due to logistical reasons, the assay could not be tested in certain patients at all the scheduled points. Although the minimum follow-up was set at post-transplant month 12, the last monitoring point was performed at month 6. In addition to budgetary considerations, the rationale for such decision was that most events would have occurred within the first 6 months, according to the classical timeline for post-transplant infection [39]. In addition, the overall amount of immunosuppression (i.e., prednisone dose and targeted trough tacrolimus levels) is usually stabilized beyond that point in most KT recipients. Therefore, it is not to be expected major changes in the results of the QTF-Monitor assay beyond month 6, as supported by the plateau between months 3 and 6 observed for IFN-γ levels (Figure 1). In addition, any conclusion on the potential usefulness of the QTF-Monitor assay for predicting bacterial or opportunistic infection should take into account that both events were considered as secondary outcomes.

In this cohort of KT recipients we found no significant association between IFN-γ production measured with the QTF-Monitor assay and the primary outcome of overall post-transplant infection. Secondary outcome analysis would suggest that the usefulness of this assay is presumably limited to the prediction of bacterial and opportunistic infection when performed within the first weeks after transplantation. Further studies are needed to establish the role of this promising method in the available repertoire of non-pathogen-specific immune monitoring biomarkers.

## Data Availability

The raw data supporting the conclusions of this article will be made available by the authors, without undue reservation.

## Abbreviations

ATG, antithymocyte globulin; auROC, area under the receiving operating characteristic curve; BKPyVAN, BK polyomavirus-associated nephropathy; CI, confidence interval; CMV, cytomegalovirus; ELISA, enzyme-linked immunosorbent assay; IFD, invasive fungal disease; IFN, interferon; IGRA, interferon-γ release assay; IQR, interquartile range; IU, international unit; KT, kidney transplantation; LT, liver transplantation; LuT, lung transplantation; NPV, negative predictive value; PRR, pattern recognition receptor; PPV, positive predictive value; QTF-Monitor, QuantiFERON Monitor; SD, standard deviation; SOT, solid organ transplantation; TLR, toll-like receptor; TTV, Torque Teno virus.
