# Supplementary material for: Performance of a Global Functional Assay Based on Interferon-γ Release to Predict Infectious Complications and Cancer After Kidney Transplantation
Source: Transpl Int. 2024 Oct 30;37:13551. doi: 10.3389/ti.2024.13551 (PMC11557340; doi:10.3389/ti.2024.13551)
Supplement: Supplementary file 1 [file DataSheet1.docx]

***Supplementary Material***

**Supplementary Methods**

*Immunosuppression and prophylaxis regimens*

Induction therapy with intravenous (IV) rabbit antithymocyte globulin (ATG-Fresenius^®^, 1.25 mg/Kg daily for 5-7 days) with delayed initiation of tacrolimus on post-transplant day 6 was used in the case of donation after circulatory death. Patients at high immunological risk also received ATG induction for 1-3 days with immediate tacrolimus initiation. Basiliximab induction (20 mg on days 0 and 4) with delayed tacrolimus introduction on day 5 was reserved to patients at risk for nephrotoxicity (i.e. older age or comorbidities). The standard maintenance immunosuppression regimen consisted of tacrolimus (0.1 mg/Kg daily, adjusted to a target trough level of 10-15 ng/mL during the first month and 5-10 ng/mL thereafter), mycophenolate mofetil (1 g twice daily) or enteric-coated mycophenolate sodium (360 mg twice daily), and prednisone (1 mg/Kg daily with progressive tapering). Conversion to mammalian target of rapamycin inhibitor (typically everolimus) with reduced-dose tacrolimus (target trough level of 3-6 ng/mL) was performed on an individual basis for recipients experiencing tacrolimus-related adverse effects, difficult-to-treat cytomegalovirus (CMV) of BK polyomavirus viremia, or malignancy.

All patients received a single IV dose of cefazolin (or ciprofloxacin in the case of hypersensitivity to ß-lactams) as preoperative antibiotic prophylaxis. Prophylaxis against *Pneumocystis jirovecii* was based on trimethoprim-sulfamethoxazole (160/800 mg three times weekly) or monthly aerosolized pentamidine (300 mg) for 9 months. Patients at high-risk for CMV infection (D+/R- or R+ with ATG induction therapy) received oral valganciclovir (900 mg daily) for 6 or 3 months, respectively. Intermediate-risk patients (R+ without T-cell-depleting therapy) were monitored every 2-4 weeks for CMV viremia with a PCR-based assay and received preemptive therapy with IV ganciclovir (5 mg/Kg twice daily) or oral valganciclovir (900 mg twice daily) for at least 2 weeks in presence of high-level (>1,000 IU/mL) or increasing viral loads.

*Additional study definitions*

Delayed graft function denoted the requirement for dialysis within the first week. Acute graft rejection was suspected in case of sudden deterioration of graft function and confirmed by graft biopsy examination. Graft loss was defined by the definitive return to dialysis, nephrectomy and/or retransplantation.

**Supplementary Results**

**Figure S1.** Correlation between IFN-γ levels as measured by the QTF-Monitor assay and (a) CD3+, (b) CD4+ and (c) CD8+ T-cell counts at month 1. IFN-γ: interferon-γ; IU: international unit.


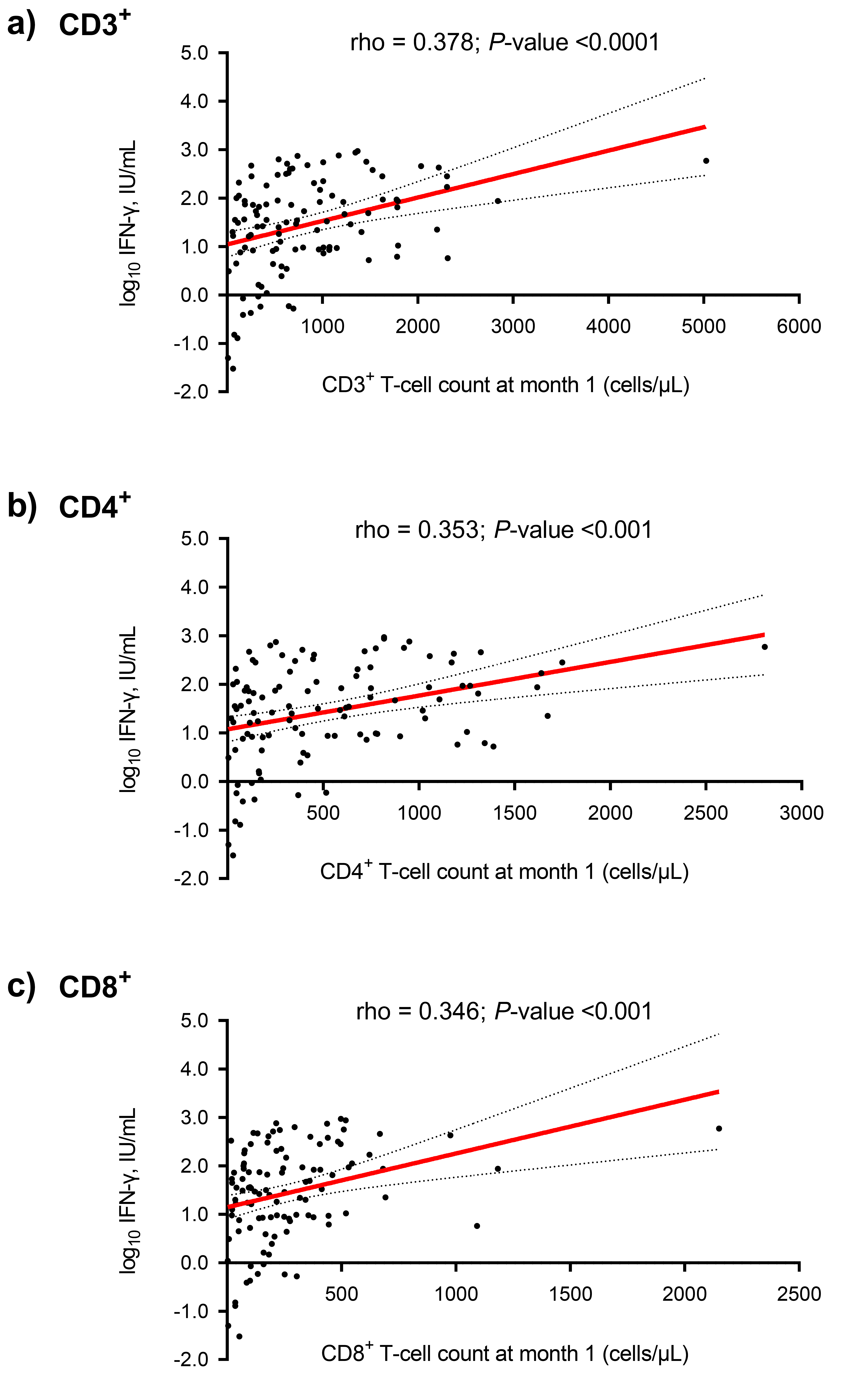


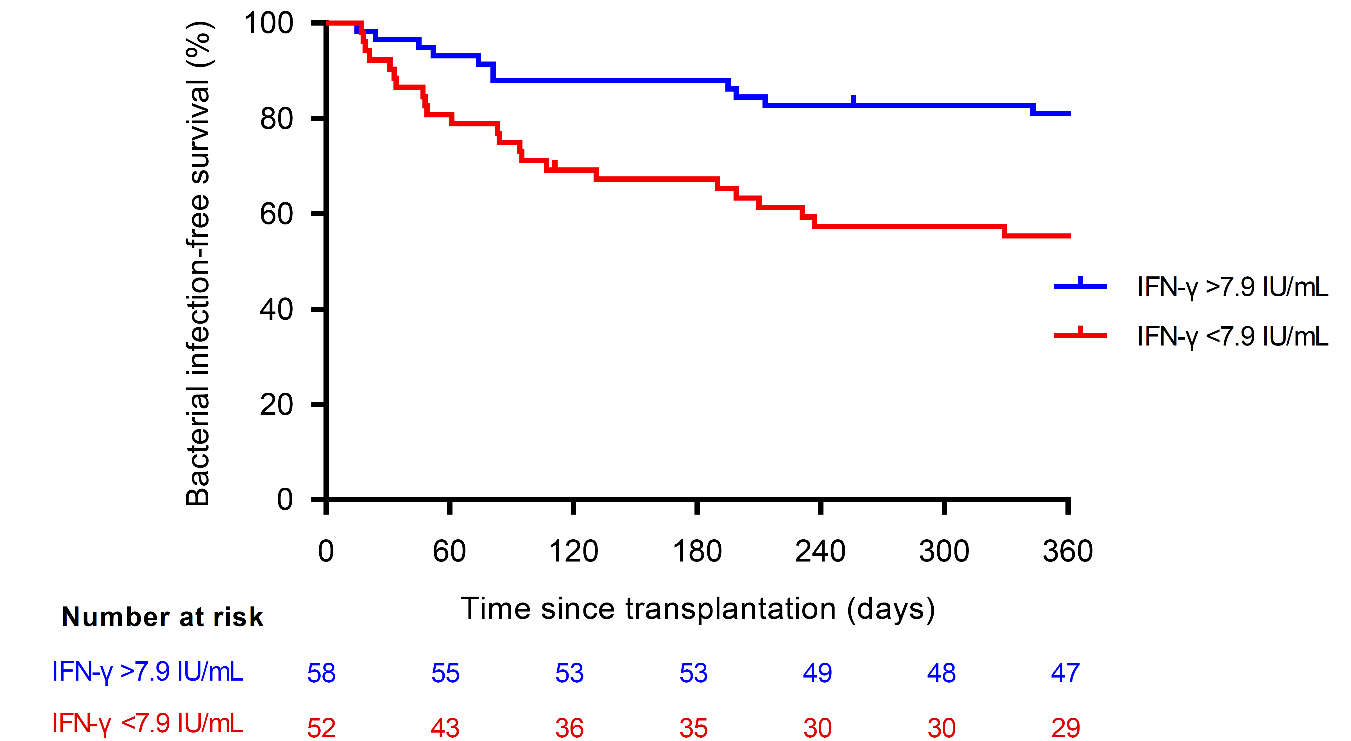
**Figure S2.** Bacterial infection-free survival according to the optimally selected cut-off value for IFN-γ level at week 2 (log-rank test *P*-value <0.001).

**Figure S3.** Opportunistic infection-free survival according to the optimally selected cut-off value for IFN-γ production at week 2 (log-rank test *P*-value = 0.005).


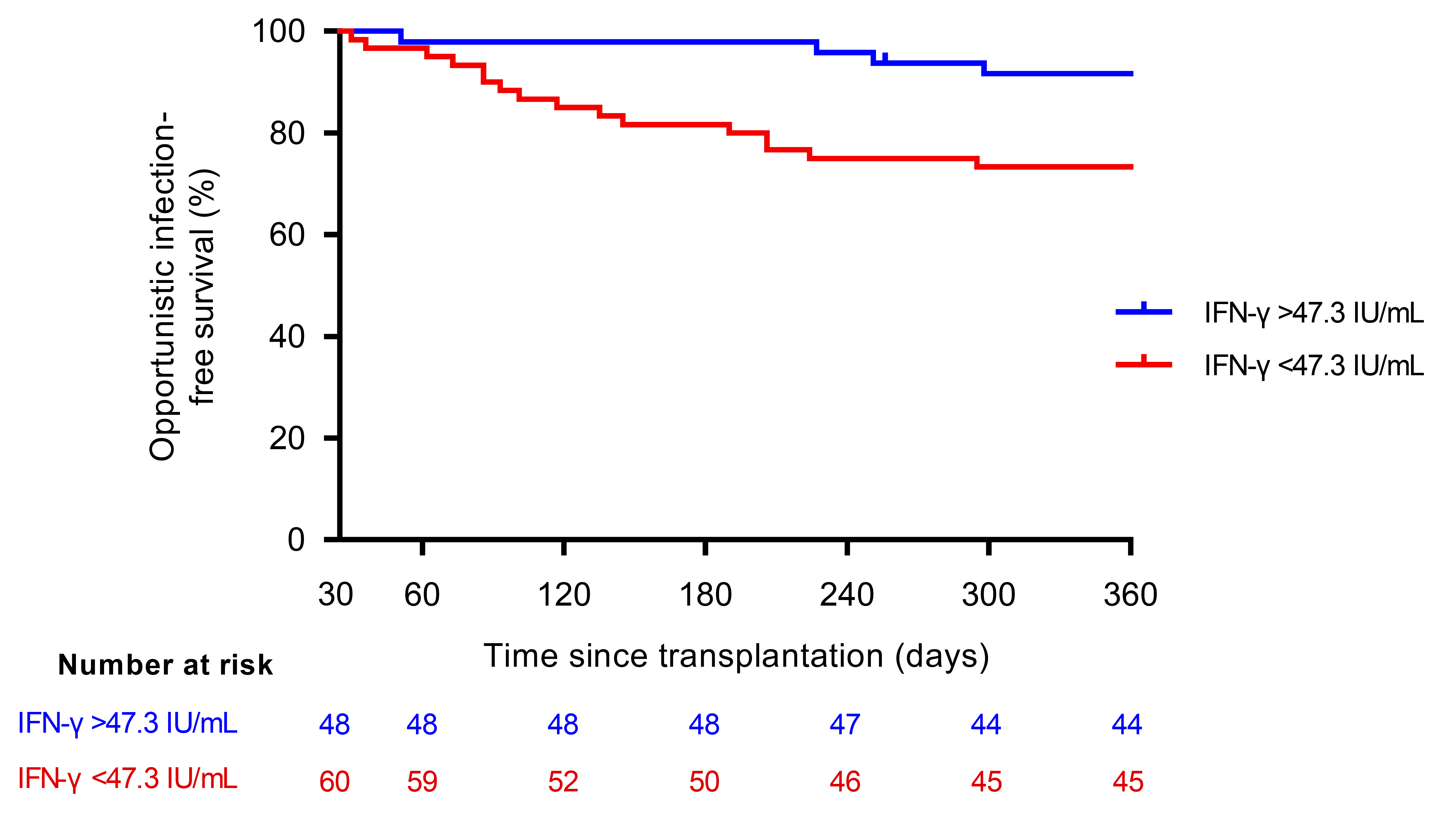


**Table S1.** Clinical determinants of low immune response (IFN-γ <15 IU/mL) in the QTF-Monitor assay performed at week 2 after transplantation.

| **Variable** | **QTF-Monitor at week 2** | | ***P*-value^a^** |
| --- | --- | --- | --- |
|  | **Low response**  (n = 66) | **Moderate-high response**  (n = 46) |  |
| Age of recipient, years [mean ± SD] | 54.9 ± 15.4 | 54.3 ± 15.9 | 0.835 |
| Male gender of recipient [n (%)] | 43 (65.2) | 29 (63.0) | 0.819 |
| Prior or current smoking history [n (%)] | 28 (42.4) | 14 (30.4) | 0.197 |
| BMI at transplantation, Kg/m^2^ [mean ± SD]^b^ | 25.1 ± 3.5 | 25.3 ± 5.3 | 0.822 |
| Diabetes mellitus [n (%)] | 21 (31.8) | 11 (23.9) | 0.362 |
| Coronary heart disease [n (%)] | 6 (9.2) | 4 (8.7) | 1.000 |
| Other chronic heart disease [n (%)] | 7 (10.9) | 7 (15.2) | 0.506 |
| Chronic pulmonary disease [n (%)] | 8 (12.1) | 3 (6.5) | 0.521 |
| Previous kidney transplantation [n (%)] | 17 (25.8) | 7 (15.2) | 0.181 |
| Glomerulonephritis [n (%)] | 14 (21.2) | 11 (23.9) | 0.736 |
| Diabetic nephropathy [n (%)] | 19 (28.8) | 9 (19.6) | 0.267 |
| Polycystic kidney disease [n (%)] | 6 (9.1) | 4 (8.7) | 1.000 |
| D+/R- CMV serostatus [n (%)] | 11 (16.7) | 10 (21.7) | 0.499 |
| Positive EBV serostatus (anti-EBNA IgG) [n (%)]^c^ | 61 (95.3) | 40 (93-0) | 0.683 |
| Positive HCV serostatus [n (%)] | 4 (6.1) | 2 (4.5) | 1.000 |
| Positive HBsAg status [n (%)] | 0 (0.0) | 3 (6.5) | 0.067 |
| Positive HIV serostatus [n (%)] | 3 (4.5) | 0 (0.0) | 0.267 |
| Pre-transplant renal replacement therapy [n (%)] | 62 (93.9) | 36 (78.3) | **0.014** |
| Time on dialysis, months [median (IQR)] | 25.9 (15.7 – 47.2) | 18 (11.6 – 46.9) | 0.217 |
| Simultaneous pancreas-kidney transplantation [n (%)] | 4 (6.1) | 0 (0.0) | 0.142 |
| Age of donor, years [mean ± SD] | 52.8 ± 17.1 | 53.9 ± 16.7 | 0.737 |
| Type of donor [n (%)] |  |  |  |
| DBD donor | 41 (62.1) | 26 (56.5) | 0.552 |
| DCD donor | 15 (22.7) | 8 (17.4) | 0.492 |
| Living donor | 10 (15.2) | 12 (26.1) | 0.152 |
| Cold ischemia time, hours [mean ± SD] | 14.8 ± 7.0 | 15.1 ± 8.1 | 0.838 |
| Number of HLA mismatches [median (IQR)] | 4 (3 – 5) | 4 (3 – 5) | 0.328 |
| Antithymocyte globulin as induction therapy [n (%)] | 39 (59.1) | 13 (28.3) | **0.001** |
| Number of doses [median (IQR)] | 4 (3 – 6) | 3 (2.5 – 4.5) | 0.094 |
| Prednisone, tacrolimus and MMF/MPS as primary immunosuppression regimen [n (%)] | 61 (92.4) | 39 (84.8) | 0.198 |
| Delayed graft function [n (%)] | 30 (45.5) | 10 (21.7) | **0.016** |
| Tacrolimus trough levels at week 2, ng/mL [mean ± SD] | 9.1 ± 3.5 | 8.1 ± 3.7 | 0.275 |
| Laboratory values at week 2 [mean ± SD] |  |  |  |
| White blood cell count, x 10^9^ cells/L | 8.9 ± 3.0 | 9.8 ± 3.9 | 0.182 |
| Absolute lymphocyte count, x 10^9^ cells/L | 0.9 ± 0.8 | 1.4 ± 0.9 | **0.005** |
| Serum albumin, g/dL | 3.8 ± 0.7 | 3.9 ± 0.6 | 0.634 |
| BMI: body mass index; CMV: cytomegalovirus; D: donor; DBD: donation after brain death; DCD: donation after circulatory death; EBV: Epstein-Barr virus; EBNA: EBV nuclear antigen; HLA: human leukocyte antigen; HBsAg: hepatitis B virus surface antigen; HCV: hepatitis C virus; HIV: human immunodeficiency virus; IQR: interquartile range; MMF/MPS: mycophenolate mofetil / enteric-coated mycophenolate sodium; R: recipient; SD: standard deviation.  ^a^ Bold characters denote *P*-values <0.05.  ^b^ Data on BMI not available for 11 and 10 patients in the low and moderate-high response groups, respectively.  ^c^ Data on EBV serostatus not available for 3 and 1 patients in the low and moderate-high response groups, respectively. | | | |

**Table S2.** Clinical determinants of low immune response (IFN-γ <15 IU/mL) in the QTF-Monitor assay performed at month 1 after transplantation.

| **Variable** | **QTF-Monitor at month 1** | | ***P*-value^a^** |
| --- | --- | --- | --- |
|  | **Low response**  (n = 38) | **Moderate-high response**  (n = 70) |  |
| Age of recipient, years [mean ± SD] | 56.7 ± 16.2 | 53.4 ± 15.1 | 0.291 |
| Male gender of recipient [n (%)] | 23 (60.5) | 50 (71.4) | 0.248 |
| Prior or current smoking history [n (%)] | 13 (34.2) | 26 (37.1) | 0.762 |
| BMI at transplantation, Kg/m^2^ [mean ± SD]^b^ | 26.4 ± 5.1 | 25.0 ± 4.0 | 0.175 |
| Diabetes mellitus [n (%)] | 15 (39.5) | 17 (24.3) | 0.099 |
| Coronary heart disease [n (%)] | 2 (5.4) | 9 (11.4) | 0.488 |
| Other chronic heart disease [n (%)] | 5 (13.5) | 7 (10.1) | 0.749 |
| Chronic pulmonary disease [n (%)] | 1 (2.6) | 8 (11.4) | 0.156 |
| Previous kidney transplantation [n (%)] | 9 (23.7) | 15 (21.4) | 0.788 |
| Glomerulonephritis [n (%)] | 7 (18.4) | 18 (25.7) | 0.391 |
| Diabetic nephropathy [n (%)] | 12 (31.6) | 14 (20.0) | 0.179 |
| Polycystic kidney disease [n (%)] | 1 (2.6) | 6 (8.6) | 0.417 |
| D+/R- CMV serostatus [n (%)] | 5 (13.2) | 16 (22.9) | 0.224 |
| Positive EBV serostatus (anti-EBNA IgG) [n (%)]^c^ | 35 (97.2) | 64 (94.1) | 0.657 |
| Positive HCV serostatus [n (%)] | 3 (7.9) | 4 (5.9) | 0.699 |
| Positive HBsAg status [n (%)] | 0 (0.0) | 4 (5.7) | 0.295 |
| Positive HIV serostatus [n (%)] | 1 (2.6) | 1 (1.4) | 1.000 |
| Pre-transplant renal replacement therapy [n (%)] | 37 (97.4) | 56 (80.0) | **0.013** |
| Time on dialysis, months [median (IQR)] | 23.3 (15.9 – 45.5) | 24.9 (12.4 – 61.2) | 0.972 |
| Simultaneous pancreas-kidney transplantation [n (%)] | 4 (10.5) | 2 (2.9) | 0.181 |
| Age of donor, years [mean ± SD] | 51.2 ± 18.8 | 53.2 ± 16.1 | 0.563 |
| Type of donor [n (%)] |  |  |  |
| DBD donor | 27 (71.1) | 40 (57.1) | 0.155 |
| DCD donor | 9 (23.7) | 11 (15.7) | 0.309 |
| Living donor | 2 (5.3) | 19 (27.1) | **0.006** |
| Cold ischemia time, hours [mean ± SD] | 15.6 ± 6.6 | 14.1 ± 7.9 | 0.325 |
| Number of HLA mismatches [median (IQR)] | 4 (3 – 5) | 4 (3 – 5) | 0.414 |
| Antithymocyte globulin as induction therapy [n (%)] | 24 (63.2) | 28 (40.0) | **0.021** |
| Number of doses [median (IQR)] | 4 (3 – 6) | 4 (3 – 5) | 0.388 |
| Prednisone, tacrolimus and MMF/MPS as primary immunosuppression regimen [n (%)] | 33 (86.8) | 61 (87.1) | 0.965 |
| Delayed graft function [n (%)] | 15 (39.5) | 21 (30.0) | 0.319 |
| Tacrolimus trough levels at month 1, ng/mL [mean ± SD] | 8.9 ± 2.8 | 9.3 ± 3.2 | 0.639 |
| Laboratory values at month 1 |  |  |  |
| White blood cell count, x 10^9^ cells/L [mean ± SD] | 7.0 ± 2.6 | 7.2 ± 2.8 | 0.791 |
| Absolute lymphocyte count, x 10^9^ cells/L [mean ± SD] | 0.9 ± 0.7 | 1.4 ± 1.1 | **0.015** |
| CD3^+^ T-cell count, x 10^9^ cells/L [median (IQR)] | 0.5 (0.2 – 0.9) | 0.7 (0.3 – 1.4) | **0.041** |
| CD4^+^ T-cell count, x 10^9^ cells/L [median (IQR)] | 0.4 (0.1 – 0.6) | 0-5 (0.1 – 0.9) | **0.037** |
| CD8^+^ T-cell count, x 10^9^ cells/L [median (IQR)] | 0.2 (0.0 – 0.3) | 0.2 (0.1 – 0.4) | 0.071 |
| Serum albumin, g/dL [mean ± SD] | 4.0 ± 0.4 | 4.2 ± 0.4 | 0.080 |
| BMI: body mass index; CMV: cytomegalovirus; D: donor; DBD: donation after brain death; DCD: donation after circulatory death; EBV: Epstein-Barr virus; EBNA: EBV nuclear antigen; HLA: human leukocyte antigen; HBsAg: hepatitis B virus surface antigen; HCV: hepatitis C virus; HIV: human immunodeficiency virus; IQR: interquartile range; MMF/MPS: mycophenolate mofetil / enteric-coated mycophenolate sodium; R: recipient; SD: standard deviation.  ^a^ Bold characters denote *P*-values <0.05.  ^b^ Data on BMI not available for 8 and 15 patients in the low and moderate-high response groups, respectively.  ^c^ Data on EBV serostatus not available for 2 patients in the low and moderate-high response groups each. | | | |

**Table S3.** Clinical determinants of low immune response (IFN-γ <15 IU/mL) in the QTF-Monitor assay performed at month 6 after transplantation.

| **Variable** | **QTF-Monitor at month 6** | | ***P*-value** |
| --- | --- | --- | --- |
|  | **Low response**  (n = 24) | **Moderate-high response**  (n = 76) |  |
| Age of recipient, years [mean ± SD] | 52.3 ± 17.3 | 54.3 ± 15.5 | 0.594 |
| Male gender of recipient [n (%)] | 18 (75.0) | 52 (68.4) | 0.540 |
| Prior or current smoking history [n (%)] | 11 (45.8) | 26 (34.2) | 0.304 |
| BMI at transplantation, Kg/m^2^ [mean ± SD]^a^ | 25.9 ± 3.9 | 25.4 ± 4.6 | 0.648 |
| Diabetes mellitus [n (%)] | 5 (20.8) | 23 (30.3) | 0.370 |
| Coronary heart disease [n (%)] | 3 (13.0) | 8 (10.5) | 0.714 |
| Other chronic heart disease [n (%)] | 2 (8.3) | 10 (13.5) | 0.725 |
| Chronic pulmonary disease [n (%)] | 1 (4.2) | 8 (10.5) | 0.683 |
| Previous kidney transplantation [n (%)] | 4 (16.7) | 16 (21.1) | 0.775 |
| Glomerulonephritis [n (%)] | 5 (20.8) | 17 (22.4) | 0.874 |
| Diabetic nephropathy [n (%)] | 4 (16.7) | 20 (26.3) | 0.335 |
| Polycystic kidney disease [n (%)] | 2 (8.3) | 6 (7.9) | 1.000 |
| D+/R- CMV serostatus [n (%)] | 4 (16.7) | 18 (23.7) | 0.469 |
| Positive EBV serostatus (anti-EBNA IgG) [n (%)]^b^ | 23 (95.8) | 71 (95.9) | 1.000 |
| Positive HCV serostatus [n (%)] | 1 (4.2) | 5 (6.7) | 1.000 |
| Positive HBsAg status [n (%)] | 0 (0.0) | 4 (5.3) | 0.570 |
| Positive HIV serostatus [n (%)] | 1 (4.2) | 1 (1.3) | 0.424 |
| Pre-transplant renal replacement therapy [n (%)] | 21 (87.5) | 66 (86.8) | 0.933 |
| Time on dialysis, months [median (IQR)] | 21.7 (15.4 – 33.9) | 23.1 (12.3 – 48.9) | 1.000 |
| Simultaneous pancreas-kidney transplantation [n (%)] | 1 (4.2) | 3 (3.9) | 1.000 |
| Age of donor, years [mean ± SD] | 56.3 ± 20.1 | 52.1 ± 16.6 | 0.310 |
| Type of donor [n (%)] |  |  |  |
| DBD donor | 16 (66.7) | 44 (57.9) | 0.444 |
| DCD donor | 3 (12.5) | 16 (21.1) | 0.551 |
| Living donor | 5 (20.8) | 16 (21.1) | 0.982 |
| Cold ischemia time, hours [mean ± SD] | 16.6 ± 7.9 | 14.2 ± 7.5 | 0.188 |
| Number of HLA mismatches [median (IQR)] | 4 (2.3 – 5) | 4 (4 – 5) | 0.228 |
| Antithymocyte globulin as induction therapy [n (%)] | 9 (37.5) | 37 (48.7) | 0.338 |
| Number of doses [median (IQR)] | 4 (3 – 4.5) | 4 (3 - 5) | 0.834 |
| Prednisone, tacrolimus and MMF/MPS as primary immunosuppression regimen [n (%)] | 23 (95.8) | 65 (85.5) | 0.176 |
| Delayed graft function [n (%)] | 10 (41.7) | 28 (36.8) | 0.671 |
| Tacrolimus trough levels at month 6, ng/mL [mean ± SD] | 9.2 ± 4.6 | 7.5 ± 2.0 | 0.116 |
| Laboratory values at month 6 |  |  |  |
| White blood cell count, x 10^9^ cells/L [mean ± SD] | 5.8 ± 2.3 | 5.7 ± 1.9 | 0.890 |
| Absolute lymphocyte count, x 10^9^ cells/L [mean ± SD] | 1.4 ± 0.9 | 1.2 ± 0.7 | 0.181 |
| CD3^+^ T-cell count, x 10^9^ cells/L [median (IQR)] | 0.9 (0.4 – 1.4) | 0.7 (0.5 – 1.1) | 0.316 |
| CD4^+^ T-cell count, x 10^9^ cells/L [median (IQR)] | 0.5 (0.2 – 0.7) | 0.4 (0.2 – 0.7) | 0.490 |
| CD8^+^ T-cell count, x 10^9^ cells/L [median (IQR)] | 0.3 (0.1 – 0.5) | 0.3 (0.1 – 0.4) | 0.587 |
| Serum albumin, g/dL [mean ± SD] | 4.5 ± 0.3 | 4.5 ± 0.3 | 0.991 |
| BMI: body mass index; CMV: cytomegalovirus; D: donor; DBD: donation after brain death; DCD: donation after circulatory death; EBV: Epstein-Barr virus; EBNA: EBV nuclear antigen; HLA: human leukocyte antigen; HBsAg: hepatitis B virus surface antigen; HCV: hepatitis C virus; HIV: human immunodeficiency virus; IQR: interquartile range; MMF/MPS: mycophenolate mofetil / enteric-coated mycophenolate sodium; R: recipient; SD: standard deviation.  ^a^ Data on BMI not available for 3 and 16 patients in the low and moderate-high response groups, respectively.  ^b^ Data on EBV serostatus not available for 2 patients in the moderate-high response group. | | | |

**Table S4.** Description of the episodes of post-transplant infection (n = 145).

| **Clinical syndrome** | **N (%)** |
| --- | --- |
| Acute graft pyelonephritis | 51 (35.2) |
| Pneumonia | 17 (11.7) |
| Upper and lower respiratory tract infection | 16 (11.0) |
| Digestive tract infection | 14 (9.6) |
| Skin and soft-tissue infection | 12 (8.3) |
| Viral syndrome | 12 (8.3) |
| Surgical site infection | 8 (5.5) |
| Intraabdominal infection | 7 (4.8) |
| BK polyomavirus-associated nephropathy | 4 (2.7) |
| Sinusitis and nasopharyngeal infections | 3 (2.0) |
| Endocarditis | 1 (0.9) |
| Isolated microorganisms | **N (%)** |
| Bacteria | 105 (72.4) |
| *Enterococcus faecalis* | 10 (6.9) |
| *Enterococcus faecium* | 3 (2.1) |
| Staphylococcus aureus | 3 (2.1) |
| Escherichia coli | 33 (22.7) |
| *Klebsiella pneumoniae* | 24 (16.5) |
| *Enterobacter* spp. | 3 (2.1) |
| *Citrobacter* spp. | 2 (1.4) |
| *Proteus* spp. | 5 (3.4) |
| Other Enterobacterales | 3 (2.1) |
| *Pseudomonas aeruginosa* | 3 (2.1) |
| *Aeromonas* spp. | 1 (0.7) |
| *Clostridioides difficile* | 7 (4.8) |
| Polymicrobial infection | 2 (1.4) |
| No microbiological diagnosis | 6 (4.1) |
| Viruses | 37 (25.5) |
| CMV | 11 (7.6) |
| HSV-1 and 2 | 2 (1.4) |
| Varicella-zoster virus | 5 (3.4) |
| BK polyomavirus | 5 (3.4) |
| SARS-CoV-2 | 6 (4.1) |
| Influenza virus | 7 (4.8) |
| Rhinovirus | 1 (0.7) |
| Fungi | 3 (2.1) |
| *Candida* spp. | 1 (0.7) |
| *Aspergillus* spp. | 1 (0.7) |
| *Pneumocystis jirovecii* | 1 (0.7) |

**Table S5.** Description of the episodes of post-transplant opportunistic infection (n = 28).

| Cumulative incidence [n/N (%)] | 26/126 (20.6%) |
| --- | --- |
| Time interval from transplantation, days [median (IQR)] | 167.5 (82.8 – 295.8) |
| Type of infection [n (%)] |  |
| CMV viral syndrome | 8 (28.6) |
| CMV end-organ disease | 4 (14.3) |
| CMV colitis | 2 |
| CMV hepatitis | 2 |
| HSV mucocutaneous infection | 3 (10.7) |
| Herpes zoster | 6 (21.4) |
| BKPyVAN | 5 (17.8) |
| Proven | 1 |
| Presumptive | 4 |
| Pneumocystis jirovecii pneumonia | 1 (3.6) |
| Invasive pulmonary aspergillosis | 1 (3.6) |
| BKPVyAN: BK polyomavirus-associated nephropathy; CMV: cytomegalovirus; HSV: herpes simplex virus; IQR: interquartile rank. | |

**Table S6.** Description of the cases of *de novo* malignancy (n = 12).

| Cumulative incidence [n/N (%)] | 11/126 (8.7%) |
| --- | --- |
| Time interval from transplantation, days [median (IQR)] | 364 (169.5 – 594) |
| Type of cancer [n (%)] |  |
| Non-melanoma skin cancer | 6 (50.0%) |
| Basal cell carcinoma | 3 |
| Squamous cell carcinoma | 3 |
| Solid cancer | 6 (50.0%) |
| Colorectal adenocarcinoma | 2 |
| Anal intraepithelial neoplasia | 1 |
| Renal cell carcinoma | 1 |
| Lung carcinoma | 1 |

**Table S7.** Cumulative incidence of secondary outcomes (bacterial infection, opportunistic infection and *de novo* malignancy) according to the immune response measured by the QTF-Monitor assay at different time points after transplantation.

| *Time point after transplantation* | **Bacterial infection** | | |
| --- | --- | --- | --- |
|  | **Low response** | **Moderate or high response** | ***P*-value^a^** |
| Incidence beyond week 2 (n/N [%]) | 33 / 65 (50.8) | 11 / 45 (24.4) | **0.006** |
| Incidence beyond month 1 (n/N [%]) | 17 / 38 (44.7) | 26 / 70 (37.1) | 0.441 |
| Incidence beyond month 3 (n/N [%]) | 5 / 15 (33.3) | 12 / 52 (23.1) | 0.504 |
| Incidence beyond month 4 (n/N [%]) | 4 / 15 (26.7) | 11 / 37 (29.7) | 1.000 |
| Incidence beyond month 6 (n/N [%]) | 4 / 24 (16.7) | 18 / 76 (23.7) | 0.469 |
| *Time point after transplantation* | **Opportunistic infection** | | |
|  | **Low response** | **Moderate or high response** | ***P*-value^a^** |
| Incidence beyond week 2 (n/N [%]) | 15 / 65 (23.1) | 9 / 45 (20.0) | 0.701 |
| Incidence beyond month 1 (n/N [%]) | 12 / 38 (31.6) | 10 / 70 (14.3) | **0.033** |
| Incidence beyond month 3 (n/N [%]) | 3 / 15 (20.0) | 6 / 52 (11.5) | 0.407 |
| Incidence beyond month 4 (n/N [%]) | 2 / 15 (13.3) | 7 / 37 (18.9) | 1.000 |
| Incidence beyond month 6 (n/N [%]) | 5 / 24 (20.8) | 8 / 76 (10.5) | 0.293 |
| *Time point after transplantation* | ***De novo* malignancy** | | |
|  | **Low response** | **Moderate or high response** | ***P*-value** |
| Incidence beyond week 2 (n/N [%]) | 5 / 66 (7.6) | 4 / 46 (8.7) | 1.000 |
| Incidence beyond month 1 (n/N [%]) | 3 / 38 (7.9) | 6 / 70 (8.6) | 1.000 |
| Incidence beyond month 3 (n/N [%]) | 1/ 15 (6.7) | 4 / 52 (7.7) | 1.000 |
| Incidence beyond month 4 (n/N [%]) | 0 / 15 (0.0) | 6 / 37 (16.2) | 0.165 |
| Incidence beyond month 6 (n/N [%]) | 1 / 24 (4.2) | 3 / 76 (3.9) | 1.000 |
| ^a^ Bold characters denote *P*-values <0.005. | | | |

**Table S8.** IFN-γ production as assessed by the QTF-Monitor assay at different time points after transplantation according to the subsequent occurrence of secondary outcomes (bacterial infection, opportunistic infection and *de novo* malignancy).

| *QTF-Monitor assay at different time points* | **Bacterial infection** | | |
| --- | --- | --- | --- |
|  | **Event** | **No event** | ***P*-value^a^** |
| IFN-γ level at week 2, log_10_ IU/mL [mean ± SD] | 0.5 ± 1.1 | 1.1 ± 0.9 | **0.002** |
| IFN-γ level at month 1, log_10_ IU/mL [mean ± SD] | 1.3 ± 1.1 | 1.6 ± 0.9 | 0.208 |
| IFN-γ level at month 3, log_10_ IU/mL [mean ± SD] | 1.8 ± 1.0 | 1.9 ± 0.8 | 0.676 |
| IFN-γ level at month 4 log_10_ IU/mL [mean ± SD] | 1.6 ± 0.9 | 1.9 ± 0.8 | 0.254 |
| IFN-γ level at month 6, log_10_ IU/mL [mean ± SD] | 2.1 ± 0.7 | 1.9 ± 0.8 | 0.771 |
| *QTF-Monitor assay at different time points* | **Opportunistic infection** | | |
|  | **Event** | **No event** | ***P*-value^a^** |
| IFN-γ level at week 2, log_10_ IU/mL [mean ± SD] | 0.6 ± 1.3 | 0.9 ± 1.0 | 0.368 |
| IFN-γ level at month 1, log_10_ IU/mL [mean ± SD] | 1.0 ± 0.9 | 1.5 ± 0.9 | **0.022** |
| IFN-γ level at month 3, log_10_ IU/mL [mean ± SD] | 1.6 ± 0.8 | 1.9 ± 0.9 | 0.247 |
| IFN-γ level at month 4 log_10_ IU/mL [mean ± SD] | 1.6 ± 0.6 | 1.9 ± 0.9 | 0.359 |
| IFN-γ level at month 6, log_10_ IU/mL [mean ± SD] | 1.8 ± 0.8 | 2.0 ± 0.7 | 0.220 |
| *QTF-Monitor assay at different time points* | ***De novo* malignancy** | | |
|  | **Event** | **No event** | ***P*-value** |
| IFN-γ level at week 2, log_10_ IU/mL [mean ± SD] | 0.8 ± 0.9 | 0.9 ± 1.1 | 0.811 |
| IFN-γ level at month 1, log_10_ IU/mL [mean ± SD] | 1.1 ± 0.8 | 1.5 ± 1.0 | 0.299 |
| IFN-γ level at month 3, log_10_ IU/mL [mean ± SD] | 2.2 ± 0.5 | 1.9 ± 0.9 | 0.445 |
| IFN-γ level at month 4 log_10_ IU/mL [mean ± SD] | 2.4 ± 0.6 | 1.8 ± 0.9 | 0.151 |
| IFN-γ level at month 6, log_10_ IU/mL [mean ± SD] | 1.9 ± 0.8 | 2.0 ± 0.8 | 0.978 |
| IFN-γ: interferon-γ; QTF-Monitor: QuantiFERON Monitor assay; SD: standard deviation.  ^a^ Bold characters denote *P*-values <0.005. | | | |

**Table S9.** Diagnostic accuracy of the QTF-Monitor assay at different positivity thresholds for IFN-γ production at week 2 and month 1 to predict protection from the subsequent development of bacterial and opportunistic infection, respectively.

| **Threshold to define “low response”** | **Proportion of patients** (%) | **Sensitivity**  (% [95% CI]) | **Specificity**  (% [95% CI]) | **PPV**  (% [95% CI]) | **NPV**  (% [95% CI]) | **PLR**  (95% CI) | **NLR**  (95% CI) | **Accuracy**  (% [95% CI]) |
| --- | --- | --- | --- | --- | --- | --- | --- | --- |
| *QTF-Monitor at week 2 to predict subsequent bacterial infection* | | |  |  |  |  |  |  |
| IFN-γ <15 IU/mL (manufacturer’s cut-off) | 58.9 | 75.0 (59.7 – 86.8) | 51.5 (38.9 – 64.0) | 50.8 (43.3 – 58.2) | 75.6 (63.8 – 84.4) | 1.55 (1.14 – 2.09) | 0.49 (0.28 – 0.85) | 60.9 (51.1 – 70.1) |
| IFN-γ <7.9 IU/mL (optimally selected)^a^ | 47.3 | 68.2 (52.4 – 81.4) | 66.7 (53.9 – 77.8) | 57.7 (47.8 – 66.9) | 75.9 (66.4 – 83.3) | 2.05 (1.38 – 3.04) | 0.48 (0.30 – 0.86) | 67.3 (57.7 – 75.9) |
| *QTF-Monitor at month 1 to predict subsequent opportunistic infection* | | |  |  |  |  |  |  |
| IFN-γ <15 IU/mL (manufacturer’s cut-off) | 35.2 | 54.6 (32.2 – 75.6) | 69.8 (58.9 – 79.2) | 31.6 (21.9 – 43.2) | 85.7 (78.8 – 90.6) | 1.80 (1.10 – 2.97) | 0.65 (0.40 – 1.05) | 66.7 (56.9 – 75.5) |
| IFN-γ <47.3 IU/mL (optimally selected)^a^ | 55.6 | 81.8 (59.7 – 94.8) | 51.2 (40.1 – 62.1) | 30.0 (24.2 – 36.5) | 91.7 (81.6 – 96.5) | 1.68 (1.25 – 2.24) | 0.36 (0.14 – 0.88) | 57.4 (47.5 – 66.9) |
| CI: confidence interval; IFN-γ: interferon-γ; NLR: negative likelihood ratio; NPV: negative predictive value; PLR: positive likelihood ratio; PPV: positive predictive value; QTF-Monitor: QuantiFERON Monitor assay.  ^a^ According to the Youden’s J statistic (J = sensitivity + specificity - 1). | | | | | | | | |
